# Annotations

**Published:** 1902-02-08

**Authors:** 


### ANNOTATIONS.

### The Incarnation of Man.

PEOPLE of a materialistic frame of mind, to whom man is but a machine, physiology but a higher kind of chemistry, and psychology but the physiology of nervous tissue—a subject subtle only because the tissue is unstable—are apt to put on one side all that cannot be weighed and measured as not only inexplicable, or, as some would say, unthinkable, but as quite beyond the range of reasonable discussion. It is a simple conception of the universe, and to many it appears to be satisfying. Let us, however, recommend such materialists, when they want a change of scene, to attend a meeting of the Society for Psychical Research. There they will find people, quite as convinced as they are of their own sanity, and quite as content as they can possibly be with the correctness of their own interpretation of things, asserting the most astounding propositions, without turning a hair. To those who are so self-centred as to think that there is something cracky about all who do not see as they do, it is a wholesome awakening to find good solid comfortable and respectable people believing in telepathy as a thing indisputable, and holding that man, as we see him engaged in his various more or less ignoble pursuits, in the city and elsewhere, is but the incarnation of one little bit of himself as he exists in an intangible and ethereal form. At the last meeting of the Psychical Research Society, Dr. Oliver Lodge, F.R.S., said that he did not hold that the whole of any one of us was incarnated in their terrestrial bodies ; certainly not in childhood ; more, but perhaps not so very much more, in adult life. What was manifested was only a definite portion of a much larger whole. What the rest was doing during the years spent here he did not know. Perhaps it was asleep, but probably, he said, it was not entirely asleep with men of genius, nor perhaps was it all completely inactive with people called mediums. Now to the modern materialist all this is absolute "rot." Yet Dr. Lodge is not exactly a man to pooh-pooh. Indeed, may not the immaterialists retort that this is a Christian country and that our very religion teaches us not to weigh and measure too exactly ? Again, Roentgen, Tesla, and Marconi have of late been giving many shocks to old ideas. At any rate, this is clear, that we must not too rigidly put outside the bounds of sanity belief in the unthinkable. It is a queer world, and which half of it is sane appears still undecided.

### The Anti-Vaccinist and the Small-pox Ships.

THERE is something peculiarly neat in the way in which the officials of the Metropolitan Asylums Board have contrived to check-mate the inquisitiveness of the National Anti-Vaccination League. According to Section 8 of the Vaccination Act every sanitary authority which maintains a hospital for the treatment of small-pox shall maintain a list of the names, addresses, ages, and condition as to vaccination of all small-pox patients treated therein, and shall at all reasonable times allow search to be made, and on certain payments being made shall supply copies. On the strength of this Mr. Corrie Grant asked the President of the Local Government Board, in the House of Commons, whether he was aware that the Metropolitan Asylums Board had repeatedly declined to supply information on the points referred to above in regard to the patients treated in their hospitals. In answer to this question Mr. Long said that the provision mentioned does not apply to the small-pox hospitals maintained by the managers of the Asylums Board, as they are not a sanitary authority within the meaning of the section. He added, however, that a register similar to that mentioned in the section is in fact kept at the hospital ships, but that only two persons have made application to inspect it. These applications appear to have been made on behalf of the National Anti-vaccination League, and the answer given to them was to the effect that arrangements would be made for the register being produced on condition that the person wishing to inspect was prepared to comply with the regulations of the managers, and satisfy the medical officer before entering the ship that he was adequately protected against small-pox. This is both reasonable and civil, but apparently there has been no great eagerness to search for knowledge at such a price. The scriptures tell us that "a soft answer turneth away wrath," but in this case it would appear that a civil answer excites wrath but turns away applicants.

### Aliens in the East End.

IN moving an amendment to the Address in the House of Commons, Major Evans Gordon put in striking form the evils which may arise from the unlimited admission of destitute aliens to this country, and Mr. Gerald Balfour in his very careful reply really added to the anxiety felt upon the subject by his gloomy prognostications as to the probable increase of this immigration in the near future. It is satisfactory to note, however, that Mr. Balfour takes hold of the right end of the question in discussing the manner in which this immigration does harm to us as a country. In his judgment he said the real evils caused by it were the insanitation and overcrowding, the enhancement of rents and the displacement of the native population, and he thought that an investigation of the subject might show that these aliens could be best dealt with, not by restrictive provisions at the port of entry, but by increasing the power of the local authorities under the Public Health Acts. Mr. S. F. Ridley (Bethnal Green) said that the slum landlord found that he could obtain twice or three times more rent for his property by letting it to the "sweater," who preyed on these destitute folk, than he could obtain from the British working man. The latter was, therefore, turned out, and his place was taken by five or eight, or even ten, families of aliens, who herded together like cattle under conditions which were at once degrading and insanitary. Here we come to the root of the matter, namely, the remissness of the sanitary authorities in not enforcing the law as to overcrowding and the insufficiency of the law itself for securing this purpose. If the aliens admitted into this country were compelled to lead a decent life, and if the "sweaters" were prevented from herding them together in insanitary conditions, this would not prevent the decent ones from coming, but it would probably be more effectual than much inspection at the ports in discouraging that wholesale importation of undesirables which is now going on.

